# Exposure to family and organized violence and associated mental health in north Korean refugee youth compared to south Korean youth

**DOI:** 10.1186/s13031-019-0230-0

**Published:** 2019-10-16

**Authors:** Jinme Park, Claudia Catani, Katharin Hermenau, Thomas Elbert

**Affiliations:** 1 Department of Psychology, University of Konstanz, Konstanz, Germany; 2 Department of Psychology, Clinical Psychology and Psychotherapy Unit, University of Bielefeld, Postbox 100131, 33501 Bielefeld, Germany

**Keywords:** Family violence, Organized violence, Trauma, PTSD, Depression, Refugee youth

## Abstract

**Background:**

Studies on populations affected by organized violence have shown elevated levels of family violence against children. This form of violence has been found to contribute to children’s psychopathology independently of traumatic experiences related to war, persecution or flight. Little is known, so far, about the exposure to family violence and its relation to mental health in North Korean refugee youth affected by political violence. The aim of this study was to examine the amount of organized and family violence and associated psychopathology in a sample of North Korean refugee youth living in South Korea compared to their South Korean peers.

**Methods:**

Sixty-five North Korean refugee youth and 65 South Korean youth were recruited. Trained researchers conducted the survey in group meetings of five to ten participants. Using questionnaires researchers assessed traumatic experiences, family and organized violence, PTSD symptoms, depressive symptoms and other mental health problems.

**Results:**

Higher rates of violence and trauma, and higher levels of mental health problems were found in the North Korean sample compared to the South Korean sample. Linear regression analyses including the various types of trauma as potential predictors showed that the severity of PTSD and depressive symptoms in the North Korean sample were associated with the amount of traumatic events and family violence but not with higher levels of organized violence.

**Conclusions:**

The findings suggest that in a context of organized violence, abusive experiences by family members constitute an important problem that is strongly linked to the psychopathology of adolescents. Our data suggest that psychological treatment and prevention approaches for North Korean refugees should be carefully tailored to fit the specific requirements of this population and address the mental health of the individual as well as potential problems at the family level.

## Background

Research on populations who have fled their homeland because of political oppression and economic hardship has consistently shown that both children and adults have been affected by a variety of adverse life experiences, including traumatic events. They have been exposed to political violence and life adversities related to poverty. The most commonly reported adversities include physical violence, torture, imprisonment, hunger, and witnessing public execution [1–3].

Adverse life experiences are not only common in a traumatic and risky life context but also seem to increase the risk of experiencing family related violence among children and adolescents. Following this line of thinking, several studies have shown a heightened prevalence of family violence in contexts of war, political violence, poverty and/or refugee status [4–8]. Based on such findings, it has been suggested that traumatic experiences have far-reaching implications not only for the individual but also for the whole family, fostering the conditions which lead to violence within the family [8]. Moreover, there is evidence suggesting that parental experiences of war and political violence are risk factors for child maltreatment [9, 10]. Therefore, children and adolescents in families affected by violent and harsh living conditions are at higher risk of experiencing violence in their home due to the deleterious effects of cumulative traumatic stress on the families.

A substantial amount of research has documented that multiple traumatic experiences increase the chances of developing Posttraumatic Stress Disorder (PTSD), which supports the presence of a dose-response effect [11]. In particular family violence has been identified as an important risk factors which contributes to a higher vulnerability for mental disorders [6, 7, 12]. Studies with children and adolescents living in insecure and violent environments have found that family violence is closely associated with a range of psychological disorders such as PTSD [7, 13–16]. There is also evidence of a link between organized violence and PTSD among children and adolescents living in traumatic and stressful environments. In a previous study on refugee minors from various countries, Mueller-Bamouh et al. [15] found that exposure to torture and war in addition to family violence was related to PTSD symptom severity.

North Korea can be regarded as a typical example of a totalitarian system where the civil population is oppressed by a military dictatorial regime. North Korean children and adolescents are therefore likely to be seriously affected by violence and poverty during the course of their life. Consistent with this assumption, North Korean refugee youth have reported multiple traumatic and violent experiences including physical violence, forced labor, imprisonment, torture, witnessing public execution, starvation, and human trafficking [1, 2, 17, 18]. Even though research on family violence among North Korean refugees is still rather limited, there is some initial evidence pointing to elevated levels of child abuse in this population [19, 20]. For instance, a study with 144 young North Korean refugees found that 59.1% of respondents reported experiences of physical or sexual violence, and 38.2% of that violence was inflicted by an immediate family member, a relative, or an acquaintance [1]. Apart from the particular political context, a combination of cultural values and parenting norms together with specific standards of child-rearing practices might also be linked to the higher levels of family violence in North Korea [21, 22]. The use of strict physical discipline methods has been persistently documented to be high in Asian families [21–24]. In fact, elevated levels of physical child maltreatment have also been reported for families in South Korea as well as in immigrant Korean families [21, 24, 25]. To date, it is not clear, whether family violence is a more common phenomenon among North Korean refugee youth compared to their South Korean peers. Even though Kim et al. [20] suggest that the incidences of child maltreatment might be higher among North Korean refugee families, there has been no direct comparison between North and South Korean samples. By including such a comparison in the present study, we sought to examine two groups who are shaped by similar cultural norms but differ with respect to their political context. So far, there is very little evidence on mental health problems and related risk factors in South Korean youth compared to North Korean samples [26, 27].

As far as the latter group is concerned, previous research has confirmed the typical dose effect relationship between traumatic experiences and PTSD [27]. However, the mechanisms behind this relationship are not fully understood. It has been suggested that the diagnosis and severity of PTSD may be linked to the diversity of experienced trauma types in addition to the amount of trauma [1, 28, 29]. Consistent with this view studies with North Korean refugees have found that PTSD is closely related to interpersonal trauma [1, 28], but not to non-interpersonal trauma [1].

Organized violence is a particular type of trauma that has been strongly associated with PTSD in North Korean samples. A number of studies suggest that the massive violations of human rights experienced by many North Koreans seem to increase the risk for psychiatric disturbances and trauma-related disorders [30–33]. The question whether exposure to family violence increases the risk for PTSD even when taking into account the level of organized violence has not been answered yet in North Korean refugee samples.

Previous studies have investigated either the relationship between PTSD and organized violence, or the association between PTSD and physical abuse. There have been no studies differentiating between family abuse, organized violence, and general traumatic events as potential risk factors for PTSD in North Korean refugee youth. The current study tries to fill this gap by including different types of traumatic adversities and determining for each of them whether it constitutes an independent risk factor for PTSD.

Taken together, the aim of the present study was, therefore, to address the links between family and organized violence, potentially traumatic events, and mental health (PTSD and depression) for both North Korean refugee youth and South Korean youth. We hypothesized that North Korean refugee youth would report more traumatic and violence experiences and higher levels of overall mental health symptoms than South Korean youth. We also hypothesized that in the North Korean sample, both family and organized violence would be identified as independent predictors for PTSD and depression symptom scores.

## Methods

### Participants

65 North Korean participants were recruited from a specialized school for North Korean refugee youth, located in Seoul. This school offers accredited high school education and commissioned junior high school education for young North Korean refugees. Every student in the 14 to 25 age group was asked to participate in the study. As a control group, we contacted three educational organizations for young South Koreans, located in Seoul. We received the permission to conduct the study from only one of the organizations, a private educational institute for youth and young adults who prepare for the college entrance examination. To be included in the study, participants had to be born in North Korea (or South Korea for the control group) and be between the ages 14 and 25. The exclusion criteria were intellectual disability, acute psychosis, current severe suicidal ideations, and drug or alcohol intoxication. With the exception of three individuals who did not meet the inclusion criteria, our overall sample consisted of 62 North Korean refugee students and 65 South Korean students. The socio-demographic characteristics of two groups are summarized in Table 1. The groups statistically differed with respect to age (t = 2.353, *p* = .022), gender ratio (*X*^2^ = 6.371, *p* = .012) and education level (Fischer’s exact test *p* < .001). The higher proportion of females among the North Korean sample is consistent with the common gender imbalance of North Korean refugees entering into South Korea (The Ministry of Unification, 2017).

**Table 1:** Sociodemographic characteristics

	North (*n* = 62)		South (*n* = 65)		*Statistical test*		
					*t*	*X* ^2^	*Fisher’s exact test*
Age (years), M (*SD*)	19.82 (range: 15–25)	2.81	18.97 (range: 18–21)	.53	2.35*	–	–
Sex, n (%)							
Male	17	27.4	32	49.2	–	6.37*	–
Female	45	72.6	33	50.8			
Education, n (%)							
Middle school	8	12.9	0	0	–	–	164.39***
High school	54	87.1	0	0			
High school graduates	0	0	65	100			
Parents alive, n (%)							
Both	31	51.7	64	98.5	–	–	41.38***
One parent only	27	45	1	1.5			
Both are not alive	2	3.3	0	0			
Household members, n (%)							
Both parents	11	18.6	54	83.1	–	–	–
One parent	28	47.5	8	12.3			
Relative/friend/NGO	12	20.3	3	4.6			
No one	10	16.9	1	1.5			
Economic support, n (%)							
Both parents	6	10	56	87.5	–	–	–
One parent	25	41.7	7	10.9			
Relatives/government/NGO	19	31.7	2	3.1			
Earn my own money	18	27.7	1	1.6			
Years since arrival in South Korea, M (*SD*)	2.9 (range: 0–9)	2.1	–	–	–	–	–
Duration of flight, n (%)							
1–4 weeks	11	18	–	–	–	–	–
2–12 months	31	50.8	–	–			
1–3 years	9	14.8	–	–			
more than 3 years	10	16.4	–	–			
Experienced forced repatriation to North Korea, n (%)							
Yes	6	10.2	–	–	–	–	–
No	53	89.8	–	–			

For Household members and Economic support, participants were able to answer multiple responses; *M* mean, *SD* Standard deviation, *p < .05; **p < .01; ***p < .001

### Procedure

All of the North Korean refugee students (*N* = 80) and South Korean students (*N* = 65) present in the respective institutions were invited to participate in this study, receiving a written invitation and informed consent form. Sixty-five North Korean students and 65 South Korean students gave informed consent to be included in the study. For participants under the age of 18 (i.e. minors by law in South Korea) an informed consent form signed by their legal guardian was required as well. Participants who consented to take part in the study arranged group appointments with the research team through their teachers. In group meetings of five to ten people, the survey was conducted under the supervision of two researchers who were trained to provide immediate psychological support if necessary. At the appointed time, each group of North Korean students gathered in a quiet room at their school. Similarly, South Korean students filled out the questionnaire in group meetings conducted in two quite rooms, outside their school.

Before the survey began, the aim and content of the study, procedure, risks, their right to withdraw and confidentiality were explained again. Only those who voluntarily signed the consent form were included in the study. Participants were then asked to answer questionnaires in Korean about family and organized violence, traumatic experiences, post-traumatic stress disorder symptoms, depressive symptoms and other mental health problems. Participants asked questions, if they did not understand the item, and the researcher provided sufficient explanation. Filling out the questionnaires required about 35 min. At the end of the survey, participants were fully debriefed and were given the opportunity to ask questions. They received financial compensation for their transportation expenses (about 8 Euro).

### Instruments

For some clinical outcomes (e.g., PTSD and behavioural problems), we used instruments developed specifically for children and adolescents, even though the sample included many young adults. We deemed this approach acceptable given that the level of education and language ability of the North Korean students did not match their actual age. The majority of them did not receive any formal education during the long process of hiding and escaping the country. The final selection of instruments was informed by discussion with local experts working with and caring for the North Korean refugee youth in South Korea. All items in the questionnaire were reviewed beforehand by teachers of North Korean youth and local mental health professionals.

#### Traumatic experiences

The trauma event checklist of the University of California Los Angeles (UCLA) PTSD Index for Children/Adolescents DSM-5 (PTSD-RI-V) [34, 35] was applied for the assessment of potentially traumatic events. The checklist consists of 14 items covering different types of traumatic events. Item four, assessing experiences of family violence, was omitted as this was already evaluated by another study instrument. The amount of exposure to potentially traumatic events was established by counting the number of different event types that were reported by the participant.

#### Family violence

Lifetime exposure to family violence was measured using the Child Version of the Parent-Child Conflict Tactics Scales (CTSPC) [36]. The Korean version of the CTSPC had been used previously in a study on child abuse and neglect conducted by the Ministry of Health and Welfare in South Korea (MOHW, 2011). The CTSPC is comprised of 27 items covering nonviolent discipline and three types of child maltreatment, psychological abuse (verbal abuse), physical assault, and neglect. The physical assaults subscale covers a wide range of severity of physical assault, and is categorized by three subscales: corporal punishment, physical maltreatment, and extreme physical maltreatment, differing in severity to the physical assaults.

Following the recommendation of the authors [36], we created a measure of physical abuse by combining the physical maltreatment and the extreme physical maltreatment subscales. This physical abuse measure also included one item from the corporal punishment subscale (slap on face or head), as this item was classified as severe physical abuse based on the judgment of experts and researchers in previous research on South Korean youth (Ministry of Health and Welfare of South Korea [MOHW], 2011).

Neglect assesses the lack or absence of adequate supervision, health care, physical care and emotional care. The CTSPC measures a prevalence score (i.e., exposure to an incident during the last year or in a lifetime) and an annual incidence score (i.e., frequency with which the incident occurred in the last year) for each item of the subscales. In the current study, the prevalence scores for physical abuse, psychological abuse and neglect were obtained based on whether the participant experienced more than one of the acts on the physical abuse, psychological abuse, and neglect subscales during their lifetime. In addition, the level of exposure to each type of family violence was calculated by summing the number of items of physical abuse, psychological abuse and neglect subscales reported by the participant, representing the total number of multiple forms of family violence the participants experienced. Cronbach’s α of the overall mean score of the CTSPC was .78.

#### Organized violence

To obtain a measure of exposure to organized violence, we generated five questions addressing political violence related to the North Korean regime. The items read as follows: “Have you seriously suffered from starvation?”; “Have you witnessed torture or public executions?”; “Have you been kidnapped or trafficked for forced labor or sexual exploitation?”; “Were you beaten up, shot at, or threatened with a knife or gun by the police or soldier?”; and “Have you been imprisoned in a prison camp or labor camp?”. The level of exposure to organized violence was calculated by the aggregate number of different event types reported by the participant. Cronbach’s α for the organized violence scale was .73 in the current sample.

#### PTSD symptoms

The prevalence and severity of probable PTSD were assessed with the UCLA PTSD Index for C/A DSM-5 [34, 35] which is a revised version of the UCLA PTSD Index for DSM-4 (UPID) [37]. The UPID is a widely-used PTSD assessment for children and young adults with good psychometric properties, which has been proven to be useful in different cultures and countries [35]. The Korean version of the UCLA Index for DSM-5 has been used previously in a study with North Korean refugee youth and reported a high internal consistency of α = .95 [38]. The new DSM-5 version consists of 27 items asking about PTSD symptoms and 4 additional items assessing the dissociative subtype. Symptom scales include criteria B (re-experiencing), criteria C (avoidance), criteria D (negative cognitions/mood), and criteria E (arousal). Participants rated the frequency of the symptoms that occurred in the past month on a 5-point Likert scale, ranging from 0 (none) to 4 (most of the time). The sum of scores on the all items of symptom scales represents the total symptom score of the UPID. In the present study, the total symptom score of the UPID was defined as the severity of PTSD symptoms. Cronbach’s α for the overall symptom score in the current sample was .96.

#### Depressive symptoms

The presence and severity of depression was measured using the Patient Health Questionnaire-9 (PHQ-9) [39]. The Korean version of the PHQ-9 has been shown to be an appropriate self-report diagnostic tool for the screening and assessment of depression both in South Koreans [40] and in young and adult North Korean refugees [41]. The PHQ-9 contains 9 items, which are rated from 0 (not at all) to 3 (nearly every day) based on the frequency of symptoms over the last 2 weeks. In the present study, the severity of depressive symptoms was defined as the sum of all 9 items (range: 0–27). Following the instructions for the PHQ-9 [42], a total score of ≥5 is regarded as indicative of probable depression. The cutoff score for considering treatment is 10. In the present study, the total PHQ-9 scores were classified into three levels of severity: abnormal (scores: 10–27), borderline (5–9) and normal (0–4) [43]. Cronbach’s α for the PHQ-9 sum score was .86 in the sample.

#### Emotional and behavioural symptoms

The self-report version of the Strengths and Difficulties Questionnaire (SDQ) [44] was used to assess emotional and behavioural symptoms. The SDQ includes five subscales covering emotional symptoms, peer problems, conduct problems, hyperactivity and prosocial behaviour. Each subscale is composed of five items that can be rated on a 3-point scale (‘not true’ = 0, ‘somewhat true’ = 1 or ‘certainly true’ = 2). Usually, items on the emotional symptom scale and the peer relationship problem scale are combined into an “internalizing behaviour” subscale, whereas the conduct problems and hyperactivity items are united in an “externalizing behaviour” subscale. In the current study, we used the sum of all items of the internalizing and externalizing subscales to generate a total difficulty score, i.e. the sum of all items of the SDQ except for prosocial behaviour. The self-report Korean version of the SDQ (the SDQ-Kr) has been reported to be highly reliable and valid for assessing emotional and behavioural symptoms in Korean children and adolescents, and the use of the total difficulties score of the SDQ-Kr was recommended for more confidence for screening [45]. Based on the cutoffs suggested by Goodman et al. [46], respondents with a total difficulty score between 20 and 40 were classified as “abnormal”, those with a score between 16 and 19 as “borderline” and those with a score below 16 as “normal”. Cronbach’s α in the current sample was .75 for the total difficulty subscale.

### Statistical analysis

Data analyses were carried out using IBM SPSS version 24.0. For dichotomous variables (i.e. the UPID total dichotomous score for trauma exposure, the CTSPC total dichotomous score, and the organized violence total dichotomous score) Chi-square tests were conducted to differences between groups. Fisher’s exact test was used to analyse group differences with respect to PTSD prevalence. For continuous variables, we used t-tests for dependent variables that were normally distributed, and Mann-Whitney U-tests for data that were not normally distributed. The data of the number of traumatic events, the PHQ score, the SDQ score was considered to be normally distributed, because the values of skewness and kurtosis were within the acceptable range of − 2 to + 2 [47, 48]. Only for the UPID score of the South Korean sample, the skewness and kurtosis values indicated a distortion of the data. Therefore, t-tests were performed to examine group differences with respect to the amount of experienced trauma types, the PHQ score, and the SDQ score. Mann-Whitney U Tests were applied to compare group differences regarding the UPID sum score as well as each of the subscale scores of the UPID. Multiple linear regression analyses with PTSD and depressive symptom score as respective outcomes were conducted separately for the North and South Korean samples to examine the associations between exposure to violence and trauma and mental health symptoms. For the North Korean sample, we entered the PTSD RI sum score and the PHQ sum score as a dependent variable, and age, the level of exposure to family violence, the level of exposure to organized violence, and the number of traumatic events as predictor variables. For the South Korean sample, more than half of the sample (58.5%) had no traumatic experience thereby the sample size was so small (*n* = 38) that we were unable to conduct a multiple regression analysis on the PTSD sum score. The regression analysis for the PHQ-9 sum score for the South Korean sample was conducted with the predictor variables age, family violence, and the number of traumatic events, excluding organized violence. In order to control for the influence of outliers, data points with high Cook’s distance (> 1) and/or with large Std. residual (> 3) were excluded. Accordingly, one data point for the North Korean sample and two data points for the South Korean sample were excluded from the analyses.

## Results

### Exposure to trauma and violence

**Fig. 1:**
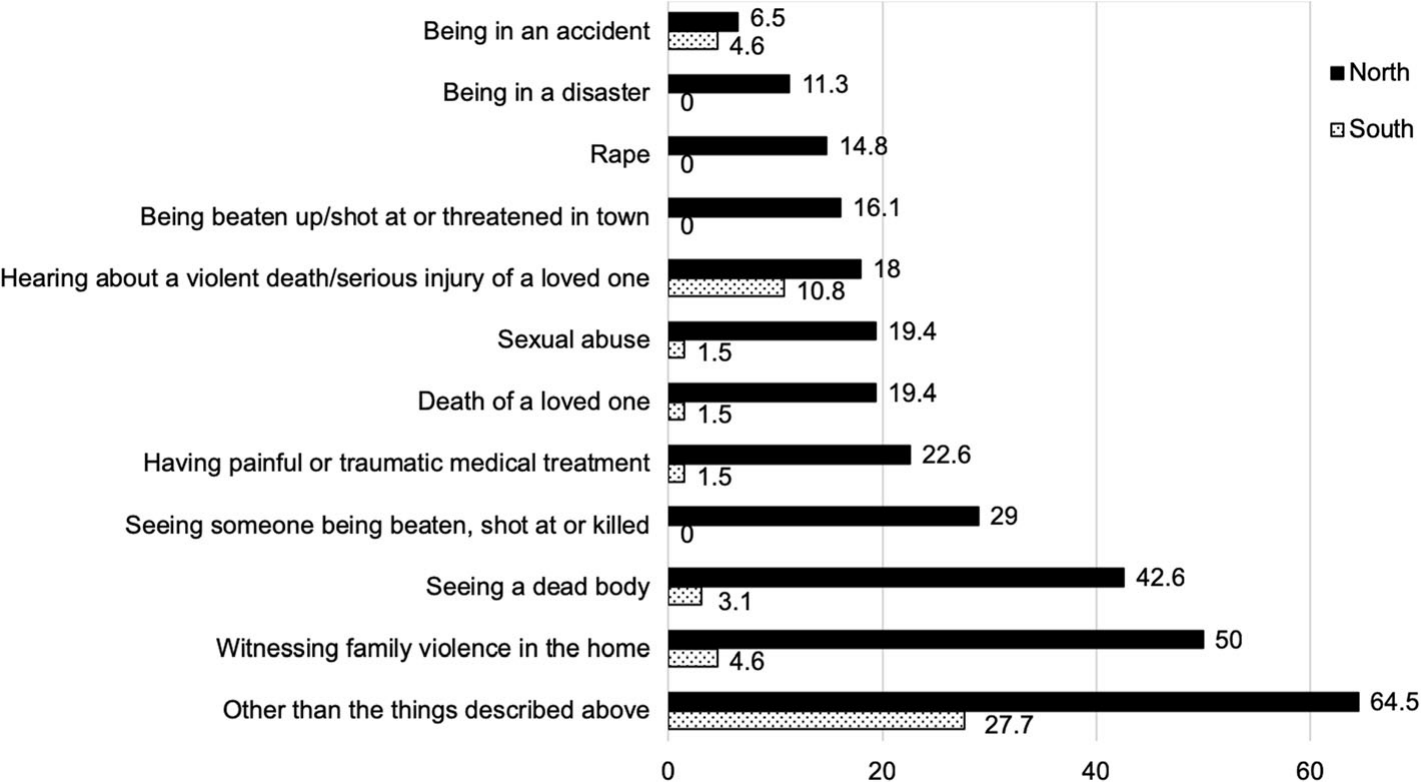
Percentages of lifetime exposure to trauma event types in the North and South Korean samples

The statistical comparison between the two groups (see Table 2) showed that exposure to traumatic events was significantly higher in the North Korean sample compared to the South Korean sample (t = 9.006, *p* < .001). 88.7% of the North Korean refugee youth had been exposed to at least one type of traumatic event that met the DSM-5 A criteria, 45.2% reported having had between two and four events and 30.6% experienced more than five traumatic events. Of the South Korean youth sample, 41.5% reported having experienced at least one traumatic event and 9.2% of the respondents experienced two or more traumatic events. The maximum number of lifetime exposure to potentially traumatic events was 8 for the North Korean sample (M = 3.13, SD = 2.11) and 4 for the South Korean sample (M = .55, SD = .81). The percentages of exposure to various types of traumatic events, based on the UPID in the two samples respectively, are presented in Fig. 1.

**Table 2:** Trauma exposure in the North and South Korean samples

	North (*n* = 62)		South (*n* = 65)		*Statistical test*	
					*t*	*X* ^*2*^
Number of traumatic events^a^, M (*SD*)	3.13	2.11	.55	.81	9***	–
Frequency of traumatic experiences^b^, n (%)						
Potentially traumatic events	55	88.7	27	41.5	–	30.86***
Family violence	35	56.5	22	33.8	–	6.56**
Organized violence	37	59.7	0	0	–	54.74***

Potentially traumatic events = exposure to traumatic events (UPID trauma checklist); Family violence = exposure to family violence (CTSPC); Organized violence = exposure to organized violence

^a^Total number of potentially traumatic events based on the UPID trauma checklist

^b^Frequency of traumatic experiences with at least one event in each type of trauma and violence, *p < .05; **p ≤ .01; ***p < .001

**Fig. 2:**
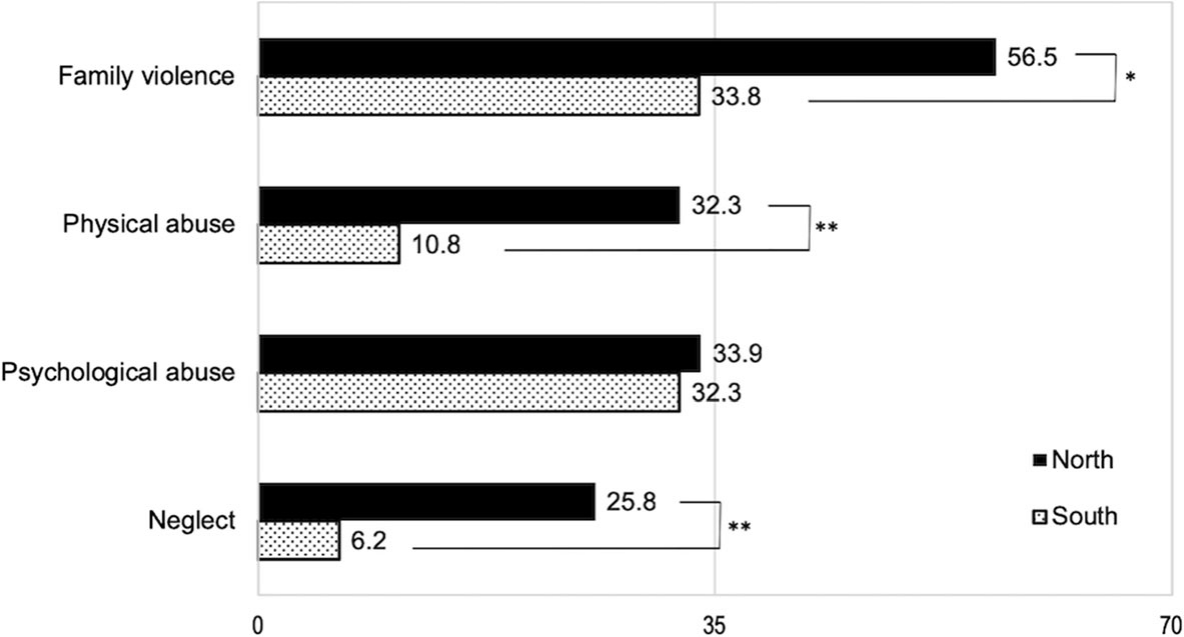
The prevalence of various forms of family violence in North and South Korean youth. Family violence = lifetime exposure to abuse and neglect (CTSPC); Physical abuse = lifetime exposure to physical abuse (CTSPC); Psychological abuse = lifetime exposure to psychological abuse (CTSPC); Neglect = lifetime exposure to neglect (CTSPC), *p < .05; **p < .01

Experiences of family violence were significantly more frequent among North Korean youth than in the South Korean sample (56.5% vs. 33.8%), *X*^2^ = 6.555, *p* = .01. 35.5% of the North Korean sample reported having experienced two or more types of family violence, whereas 23% of the South Korean sample reported two or more types. Figure 2 illustrates the lifetime prevalence of various forms of family violence in the two samples. Compared to the South Korean sample, the North Korean sample had significantly higher rates of physical abuse (32.3% vs. 10.8%), *X*^2^ = 8.753, *p* = .003, and neglect (25.8% vs. 6.2%)*, X*^2^ = 9.237, *p* = .002, whereas psychological abuse did not differ between the two groups.

Exposure to organized violence was reported by more than half of the North Korean refugee youth in our sample (59.7%), whereas no one has been exposed to this type of violence in the South Korean youth sample. The mean number of exposures to organized violence for the North Korean sample was 1.19 (SD = 1.33), with a maximum number of five different incidences linked to organized violence. The three most frequent types were suffering from starvation (43.5%), witnessing torture or public execution (27.4%), and imprisonment (21%).

### Mental health outcomes

**Fig. 3:**
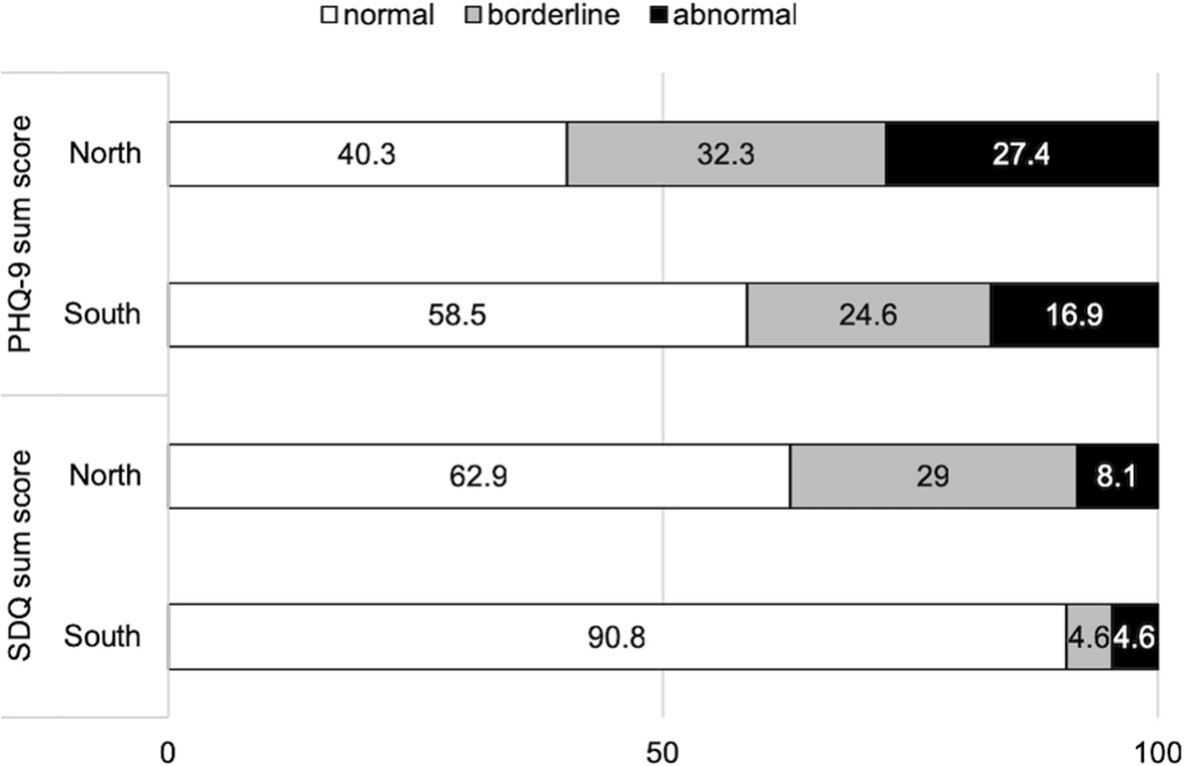
Frequencies (%) of critical scores on the PHQ-9 and the SDQ in the samples, respectively. PHQ-9 sum score = depressive symptoms; SDQ sum score = the total difficulty symptoms

Table 3 provides an overview on group means and statistical differences between the two samples with respect to the standardized clinical questionnaires. Compared to their South Korean peers, North Korean refugee youth reported significantly higher levels of PTSD symptoms (Mann-Whitney U = 800, *p* < .001), and emotional and behavioural symptoms (t = 4.702, *p* < .001). With respect to depressive symptoms, the mean score of the PHQ-9 for the North Korean sample (M = 6.87, SD = 5.58) was higher than that of the South Korean sample (M = 5.18, SD = 5.45), however the difference reached only a trend-level of significance (t = 1.722, *p* = .087). Figure 3 shows the frequencies of critical scores on the PHQ-9 and the SDQ, separately for each group. The North Korean sample showed more emotional and behavioural problems compared to the South Korean sample (Fischer’s exact test *p* < .001), whereas the amount of borderline to abnormal depression scores on the PHQ-9 did not differ between the two groups (*X*^2^ = 4.344, *p* = .113).

**Table 3:** Comparison of mental health problems between the North and South Korean samples

	North (*n* = 62)		South (*n* = 65)		*Statistical test*		
					*Fischer’s exact test*	*Mann-Whitney U*	*t*
PTSD prevalence, n (%)	8	12.9	1	1.5	6.23*	–	–
UPID sum score, M (*SD*)	17.06	17.96	3.62	8.9	–	800***	–
re-experiencing	3.63	5.01	.59	2.2	–	1034***	–
avoidance	1.76	2.47	.64	1.47	–	1357**	–
negative cognitions/mood	6.71	6.68	1.40	3.38	–	826***	–
arousal	4.97	5.28	1.02	2.57	–	924***	–
PHQ-9 sum score, M (*SD*)	6.87	5.58	5.18	5.45	–	–	1.72
SDQ sum score, M (*SD*)	13.66	5.28	9.52	4.63	–	–	4.70***

UPID sum score = PTSD symptom severity; PHQ-9 sum score = depressive symptom severity; SDQ sum score = the severity of total difficulties; *M* Mean, *SD* Standard deviation, *p < .05; **p < .01; ***p < .001

### Relationships between trauma exposure and mental health

Table 4 provides an overview on the findings of the linear regression models on the amount of PTSD and depressive symptoms in the North Korean sample. For both outcomes, the level of exposure to family violence and the number of traumatic events resulted as significant predictors. Regarding the South Korean sample, the regression model on depressive symptoms did not reach statistical significance (adj R^2^ = .000, F = 1.001, *p* = .399), therefore, the results are not presented here.

**Table 4:** Predictors of PTSD and depressive symptoms in the North Korean sample

Predictor	UPID sum score		PHQ-9 sum score	
	North (*n* = 62)		North (*n* = 62)	
	ß	r	ß	r
Age	−.06	−.06	−.08	−.20
Number of traumatic events	.33*	.44	.32*	.32
Level of exposure to family violence	.27*	.33	.48***	.54
Level of exposure to organized violence	.12	.31	−.11	.08

adj*R*^*2*^ (UPID sum score) = .220, *F* = 5.226, *p* < .01, adj*R*^*2*^ (PHQ-9 sum score) = .318, *F* = 7.999, *p* < .001., UPID sum score = PTSD symptom severity; PHQ-9 sum score = depressive symptom severity; Number of traumatic events = the total number of potentially traumatic events (UPID checklist); Level of family violence = the number of lifetime exposure to family violence (CTSPC); Level of organized violence = the number of lifetime exposure to organized violence, *p < .05; ***p < .001

## Discussion

The present study found higher rates of violence and trauma, and higher levels of mental health problems in the North Korean sample compared to the South Korean sample. A crucial finding was that both the experience of family violence and trauma exposure were significant predictors of PTSD and depressive symptom severity in the North Korean sample.

The great majority (88.7%) of the North Korean sample reported having experienced a traumatic event whereas this was the case only for 41.5% of the South Korean youth. Around 60% of the North Korean sample reported having experienced organized violence. The prevalence of incidences of family violence in the North Korean sample (56.5%) was significantly higher compared to the South Korean sample (33.8%), but similar when compared to the rates in refugee families from other contexts [49, 50]. Therefore, the present finding is in line with previous research supporting a link between organized violence and elevated rates of family violence among conflict-affected populations [5, 7, 9]. In this regard, Timshel et al. [8] suggest that the likelihood of exposure to violence home increases as multiple risk factors accumulate at the individual, familial, societal, and cultural levels. We might therefore assume that the cumulative experiences of political oppression and trauma in the North Korean sample could be one reason for the more frequent experiences of family abuse. Moreover, next to culture norms, national and regional policies including child abuse prevention advocacy may have an impact on the use of physical discipline methods towards children [22]. Even though parental discipline was legally allowed, and there was no explicit prohibition of physical punishment in South Korea at the time of the study [51, 52], the promotion of non-violent child rearing strategies in Western countries may have had a larger impact on parents in South Korea than those in North Korea, who are kept mostly isolated from western influences. In fact, just recently in 2019 the Korean government has accepted recommendations of the UN Committee on the Rights of the Child, and has advocated the prohibition of corporal punishment [51]. In contrast, according to the Human Rights Watch’s World Report 2018 and progress reports by the Global Initiative to End All Corporal Punishment of Children, the little information available on North Korea suggests that children’s rights are not protected and that violence against children, whether within or outside the family, is not punished [51–53]. Considering this line of reasoning, our finding that specifically physical abuse as opposed to psychological abuse was higher in the North compared to the South Korean sample seems plausible.

Consistent with findings from a previous study [26], the North Korean sample presented higher levels of PTSD and depressive symptoms as well as a higher amount of emotional and behavioural symptoms compared to the South Korean sample.

Regarding the role of family abuse in the prediction of posttraumatic stress, we identified the amount of family violence, next to general traumatic events, as a significant predictor for PTSD and depressive symptoms in the North Korean sample. This is in line with previous studies suggesting that the experience of family violence increases the risk for PTSD and other mental health problems in populations affected by political conflict and violence [5, 6, 15, 16]. Our finding supports not only earlier evidence of a dose-response relationship between trauma exposure and mental health problems in people who fled North Korea [27], but also points towards family violence as an independent risk factor associated with PTSD and depression.

The amount of organized violence was not included as a specific predictor of either PTSD or depressive symptoms. This finding is incongruent with previous studies showing a close link between organized violence and PTSD [15, 54, 55]. Two possible explanations could account for this discrepancy. First, the assessment of organized violence with only five items in the present investigation was not comprehensive enough. In fact, other studies employing standardized checklists to assess the amount of events related to conflict and persecution typically reported higher incidences of organized violence [15, 54]. Second, we did not assess torture as a specific type of organized violence. Previous studies assessing more specifically different types of organized violence have consistently reported a strong connection between PTSD and torture experiences [15, 54–56].

It is worth noting that we found an increased occurrence of clinically relevant depressive symptoms (16.9%) also in the South Korean sample that did not statistically differ from the rate in the refugee group. However, we were unable to determine risk factors associated specifically with depressive symptoms in the South Korean sample. One reason for this might be that, due to the small sample size, we were not able to include the different types of adverse childhood experiences in the regression analyses. In fact, our results show that psychological abuse was the only type of family violence that was reported similarly frequently in both groups. There is evidence from previous research on the long-term consequences of childhood maltreatment showing that, compared to physical or sexual abuse, psychological or emotional abuse is more strongly linked to depression [57–59]. Thus, the relatively high amount of clinically relevant depression symptoms in the South Korean sample might be related to the frequent reports of childhood psychological violence by family members.

In addition, it might be speculated that there are important factors associated with depression other than family violence and traumatic life events that have not been assessed here. In fact, there are studies showing that depressive symptoms are linked to poor school performance and/or peer problems in South Korean adolescents [60–62]. Related to this, Kim and Shin [26] found that South Korean adolescents reported higher scores on academic stress compared to North Korean adolescent defectors. Unfortunately, the present study did not include the assessment of academic achievement, so further research would be needed to clarify any potential association with depression.

There are more limitations that should be taken into account when discussing the present findings. First, our sample size was small and the North Korean refugee sample examined in the study was comprised mostly of females, resulting in a gender imbalance between the samples. In addition, the South Korean sample differs from the North Korean sample with respect to education level, age and gender so that group differences cannot be attributed solely to the different political context. However, there are studies with South and North Korean students indicating that mental health is not necessarily affected by education level or gender [26, 63]. For instance, previous research with South Korean youth [63] did not find any differences between high school and middle school students with respect to depression, anxiety, aggressive behaviour and conduct problems. In addition, aggressive behaviour and conduct problems did not differ between male and female participants [63]. We might therefore assume, that the differences found between the North and South Korean samples in the present paper cannot be entirely attributed to age and gender. As a second drawback of our study, it should be mentioned that measures for PTSD symptoms and organized violence have not been validated specifically for the Korean context. However, the rate of probable PTSD of the North Korean sample in the present study is similar to that obtained in comparable previous studies [1, 64] using the Posttraumatic Stress Diagnostic Scale (PDS) [65]. Third, although it was included in the list of potentially traumatic events, we did not specifically focus on sexual abuse which is well known to be associated with the development of mental disorders [66]. Thus, larger studies using more comprehensive and detailed measurements of family and organized violence are required.

## Conclusions

Although our finding should be interpreted with caution, this study is the first attempt to compare exposure to trauma and violence as well as mental health problems between North Korean refugee youth and South Korean youth. The present study also contributes to a better understanding of exposure to trauma and violence and its relation to mental health problems in young people who fled North Korea. It could be shown that a considerable percentage of North Korean refugee youth have experienced multiple types of family violence in addition to organized violence and general traumatic events, which place them at higher risk for PTSD and depression.

The findings of the present study have implications for the development of diagnostic and psychosocial treatment services that should be offered to North Korean adolescents who reach South Korea. Next to individual trauma treatment for adolescents diagnosed with PTSD, there seems to be an additional need for intervention and prevention programs at the family level developed to end or prevent violence in the family and foster positive parenting strategies. On an even broader level, psychoeducational programs could be useful to raise awareness and educate the refugee youth, caregivers and teachers about family violence and its consequences in the context of persecution and flight. Given that the psychological problems faced by North Korean refugees are linked to a lower quality of life and difficulties in adapting to South Korea [67], specifically tailored mental health care services for North Korean refugee youth will not only improve the mental health but might also promote social inclusion.

## Data Availability

The data that support the finding of the current study are available from the corresponding author, CC, on reasonable request.

## Abbreviations

CTSPC: Child Version of the Parent-Child Conflict Tactics Scales
MOHW: Ministry of Health and Welfare in South Korea
PHQ-9: Patient Health Questionnaire-9
PTSD: Posttraumatic stress disorder
SDQ: Strengths and Difficulties Questionnaire
UPID: University of California Los Angeles PTSD Index for DSM-4
